# Supporting Patients’ Use of Digital Services in Primary Health Care in England: Synthesis of Evidence From a Mixed Methods Study of “Digital Facilitation”

**DOI:** 10.2196/52516

**Published:** 2024-12-04

**Authors:** Jon Sussex, Helen Atherton, Gary Abel, Christopher Clark, Emma Cockcroft, Brandi Leach, Christine Marriott, Jennifer Newbould, Emma Pitchforth, Rachel Winder, John Campbell

**Affiliations:** 1 RAND Europe, Eastbrook House, Shaftesbury Road, Cambridge, CB2 8BF, United Kingdom, 44 1223353329; 2 University of Southampton, Southampton, United Kingdom; 3 University of Exeter Medical School, Exeter, United Kingdom; 4 National Institute of Health and Care Research Collaboration South West Peninsula Patient Engagement Group, University of Exeter Medical School, Exeter, United Kingdom

**Keywords:** web-based health services, primary care, digital facilitation, evidence synthesis, medical practitioners, digital services, digital intervention, mixed methods study, scoping review, ethnography

## Abstract

**Background:**

General medical practitioners and other staff at primary care medical practices have an important role in facilitating patient access to online services in the National Health Service in England. These services range from online ordering of repeat prescriptions to conducting online consultations with health care professionals. We have defined “digital facilitation” as that range of processes, procedures, and personnel that seeks to support patients in their uptake and use of online services.

**Objective:**

We report how we have synthesized the evidence from a mixed methods study of digital facilitation in primary care in England. The study’s objectives were to identify, characterize, and explore the benefits and challenges of different models of digital facilitation in general medical practices in England and to design a framework for evaluation of the effectiveness and costs of digital facilitation interventions.

**Methods:**

Our study comprised scoping review of literature, survey of staff in general practices, survey of patients, and ethnography at case study practices plus stakeholder interviews. We compiled a triangulation matrix of the findings from individual work packages through an iterative process whereby each work package’s results were first analyzed separately and were then cumulatively combined across work packages in 3 successive workshops. From the resulting matrix, we developed a program theory and an implementation theory and constructed a framework for evaluations of digital facilitation in primary care. The final step of the synthesis process was to discuss the results with national and regional National Health Service stakeholders.

**Results:**

Triangulation yielded a combined set of findings summarized within 11 thematic groupings: 3 setting the scene within which digital facilitation takes place, and 8 related to different types of digital facilitation, their implementation, and effectiveness. Some thematic groupings were evident in the findings of all 4 of the research work packages; others were not addressed in all the work packages but were evident from those where they were addressed. Throughout the synthesis, there were no instances where findings from one work package contradicted the findings of another. Findings either reinforced each other or offered complementary or additional insights. The discussion at the stakeholder meeting held at the end of the study resulted in the research team clarifying some findings but not changing any of them.

**Conclusions:**

Digital facilitation can take many forms, though much of what is currently done in primary care practices in England is reactive and passive. Clear lines of responsibility, digital tools and platforms that work well for patients and practice staff, and investment in staff time and training are all needed if digital facilitation is to deliver on its promise. We propose a framework for future evaluations of the effectiveness and costs of digital facilitation interventions.

## Introduction

The National Health Service (NHS) offers all UK residents primary care physician services delivered by general medical practitioners (GPs). GPs work in local practices in multidisciplinary teams with nurses and other health care professionals such as health care assistants, pharmacists, and paramedics, all supported by staff and administrative and reception staff. Each individual patient registers with a GP practice of their choice, usually near to where they live, and the practice is funded via a capitation fee and other payments from the NHS; GP services are free of charge to patients. That practice is responsible for their registered patients’ care. GPs and other primary care staff consequently have an important role in facilitating access to the growing range of digital NHS services. It is crucial to note that colloquially within the health service in England, patient-facing services that use the web are known as “online services” [1] but that at policy level and in the academic literature, these services are among what are referred to as “digital” services [2], with digital used as an umbrella term. As a consequence, we use the 2 terms within this paper applying the term “online” when referring to the use of services within general practice in the NHS in England, and “digital” to refer to the wider context.

This paper reports how we synthesized the evidence from multiple parts of a mixed methods study of such “digital facilitation” in NHS primary care in 4 regions of England [3] and the overall findings of that synthesis.

Health care policy in England, in common with many other countries, highlights the role and potential of digital services for patients. There have been increasing contractual requirements for GPs to offer and promote a range of online services following the publication of the NHS Long Term Plan [4] and associated 5-year framework for GP contract reform [5] in 2019. Online services in primary care range from ordering repeat prescriptions, viewing test results, and booking appointments with primary care professionals, to online consultations between patients and doctors. There is considerable diversity in the online services offered across more than 6000 general practices in England. The move to increase reliance on online means of accessing services stems from assumptions that they improve choice, convenience, and ease of access for users; improve triage systems; and streamline service delivery [6-8]. The exigencies of the COVID-19 pandemic from March 2020 onward reinforced and accelerated this initiative [9]. Increased emphasis on many types of digital services has occurred in may jurisdictions [10], associated with the development of policy responses to support the continuing use and growth of such services. In New Zealand, for example, there has been recognition that a longer-term strategy is needed to provide comprehensive and less fragmented digital services in primary care [11].

Offering digital services is not enough to ensure that people use them. Patients may need encouragement and help engage with and use digital services. Our research addresses the experience and future potential of digital facilitation by GPs and other practice staff (ie, providers of primary care). We have defined “digital facilitation” as that range of processes, procedures, and personnel, which seeks to support patients in their uptake and use of online services. This is a pragmatic definition intended to include actions that specifically support patients to take up and use online services already available within the NHS. Although our study focuses on primary care in England, it has relevance for any health care system offering digital services to patients. A scoping literature review undertaken as the first stage of our research found international evidence that digital facilitation can be effective [12].

Our research had 2 principal aims overall: first, to identify, characterize, and explore the potential benefits and challenges of different models of digital facilitation in use in NHS primary care practices in England; and second, to design a framework for future evaluations of the effectiveness and costs of such interventions.

## Methods

### Overview

We conducted four main, interrelated research work packages over the period 2020‐2022: (1) a scoping review of literature [12] to determine the types of digital facilitation relevant to primary care, their effectiveness, and cost-effectiveness; (2) a survey of staff in 156 general practices in 4 regions of England: East of England and North London, North-West, South-West, and West Midlands; (3) a survey of 3051 patients from a sample of general practices in East of England and North London, South-West, and West Midlands; and (4) qualitative exploration, based on rapid ethnography undertaken at 8 case study practices and 19 additional interviews with senior stakeholders, to understand in-depth and from the perspective of staff, patients, and other stakeholders the benefits and challenges of different models of digital facilitation. Full details of the methods and findings of each work package are presented elsewhere [3]. This paper focuses on how we have synthesized the evidence from the 4 work packages and the implications of that synthesis.

We have used as our theoretical framework Weiss’s approach to theory-based evaluation [13]. The essence of our approach was to compile a triangulation matrix [14] of the findings from individual work packages. The matrix was developed in an iterative process that exploited the staggered completion of the different work packages. First, the researchers for each work package analyzed their findings. The work package findings were then brought together and analyzed cumulatively in a series of 3 half-day workshop meetings of researchers from the relevant work packages and members of the public. Two of the researchers who participated in all 3 workshops were part-time staff in general practices, 1 as a GP (JC) and 1 as an administrator (RW). Among the other participants were members of the patient advisory group established for the research project. Briefing material was circulated to all participants in advance and plenty of time was allowed at the start of each workshop for discussion and clarification of that material. In each workshop the participants identified themes arising from the developing data set and discussed how different sources of evidence reinforced, added to, or differed from one another in their findings.

The first synthesis workshop took place online in November 2021. Nine researchers and 3 patient advisory group members analyzed the findings from the practice survey and the scoping literature review. At the second synthesis workshop in July 2022, also online, 12 researchers and 3 patient advisory group members added the emerging findings from the qualitative research. The final synthesis workshop was held in-person in September 2022 and involved 15 researchers and 3 patient advisory group members, bringing together the entire evidence set from all work packages. The output from the third workshop was an agreed set of 11 thematic groupings identified from the evidence.

Following Weiss’s approach [13], we used this knowledge to develop a “program theory,” which specifies the mechanism of change, and an “implementation theory” of how digital facilitation is operationalized and what facilitates or hinders its implementation. From the 11 thematic groupings, the program theory and implementation theory, and with reference to UK Medical Research Council guidance on evaluating complex interventions [15], we then constructed a framework for future evaluations of digital facilitation approaches in primary care.

The final step of the synthesis process was to discuss the themes, program and implementation theories, and evaluation framework at an online meeting in December 2022 with representatives of NHS stakeholders at national and regional levels. The participants were from the national body responsible for the operation of the NHS in England (NHS England); the national body specifically responsible for implementation of digital health care services (a part of NHS England); and one of the 42 integrated care boards, which are NHS bodies responsible for commissioning and coordinating health services at a regional level. We shared findings and invited the challenge and review of participants, considering whether change in findings, interpretation, or emphases was warranted.

### Ethical Considerations

Ethics approval was obtained for the patient survey and ethnographic case studies within the research project from the North East—Newcastle and North Tyneside 2 Research Ethics Committee on April 27, 2021. Health Research Authority approval was obtained in July 2021 (IRAS number 289425, protocol number L01886). All methods were carried out within the ethical and data governance guidance overseeing this project. This research was carried out in compliance with the World Medical Association Declaration of Helsinki. Ethics approval was not required for the practice survey element (as advised by the Health Research Authority) because the survey did not intend to change practice or patient care. Patients were deemed to have consented to participate in the patient survey if they returned a questionnaire either by post or online (implied consent). The research team did not ask for any personal data from survey participants, although participants could provide their contact details (which were kept separate from other survey data) if they wished to take part in the prize draw. Information on processing of personal data on the participant information sheet provided an explanation of our approach to handling personal data. Practices responding to the practice survey were entered into a prize draw for 1 of 10 £250 (US $316) vouchers. A voluntary prize draw for 1 of 10 £25 (US $32) vouchers was offered as an incentive for patients participating in the patients survey. Potential patient survey respondents were informed that consent would be assumed upon return of a questionnaire either by post or online. Analysis of General Practice Patient Survey data was deemed service evaluation not requiring ethics approval.

## Results

### Themes

#### Overview

The detailed results from the literature review, practice survey, patient survey, and qualitative research are reported elsewhere [3,12]. Triangulation of the results from these sources yielded a combined set of findings summarized within 11 thematic groupings: 3 of them setting the scene within which digital facilitation takes place and 8 related to different types of digital facilitation, their implementation, and their effectiveness. Some of the themes (we use this shorter notation from here on) were evident in the findings of all 4 of the research work packages. Other themes were not addressed in all the work packages but were evident from 1, 2, or 3 of them. Throughout the synthesis work, there were no instances where findings from one work package contradicted the findings of another. Findings either reinforced each other or offered complementary or additional insights. The discussion at the stakeholder meeting at the end of the study resulted in the research team clarifying some of its findings but not changing any of them.

The 11 themes are described in turn in the following paragraphs. We first present the 3 scene-setting themes. These were identified from the qualitative exploration. Thus, they emerged from the discussion in the second synthesis workshop—when the emerging results of the qualitative exploration were added into the evidence synthesis process. The third scene-setting scene, related to COVID-19, had also been implicit in some of the practice survey data that reflected the major changes that primary care underwent in response to the pandemic, including reductions in face-to-face consultations and increased reliance on remote methods of ordering prescriptions, for example. The sources that led to the generation of the other themes are explained for each theme in turn.

##### Theme 1 (Scene Setting)

The *value and purpose of digital services* determines the usefulness of facilitating use of those services. Our qualitative research found that the value of some digital services, and what they are expected to achieve, is not always clear to primary care staff or patients and views and understanding about the value and purpose of such services may differ.

##### Theme 2 (Scene Setting)

*“Digital” conflates online with other routes to access* primary care. The qualitative research showed that while patients are greatly interested in navigating the system to gain access to health care, the distinction between “online services” and other access routes may not be important to them. Indeed, any way of accessing primary care remotely is sometimes seen by patients as “digital,” including telephone consultations.

##### Theme 3 (Scene Setting)

Our survey of GP practices confirmed that the *COVID-19* pandemic and the measures taken in response to it had led to major changes in primary care, including much-increased reliance on remote ordering of prescriptions as well as remote consultations. The qualitative research at case study sites found that at the same time as the need for digital facilitation increased, the pandemic led to the cessation of some digital facilitation initiatives (such as using tablet computers to sign patients up to online services while they sat in the practice waiting room).

##### Theme 4

*Defining and identifying digital facilitation*: at the beginning of the study, the research team had defined “digital facilitation” as stated earlier and based the literature review on this, but practice staff may have different definitions. The practice survey, considered with the literature review findings at the first synthesis workshop, revealed that in some practices ad hoc facilitation efforts, such as receptionists answering queries from patients about using online services, would not be seen as digital facilitation but in other practices they would be. The patient survey and the qualitative research then added to this theme by showing that patients may not be aware of digital facilitation per se.

##### Theme 5

*Types of digital facilitation* can be active or passive, reactive, or proactive. This was already clear after the literature review and hence the range of types of digital facilitation was already an emergent theme at the first of the 3 synthesis workshops. We revisited the typology at each subsequent synthesis workshop. An example of active digital facilitation is a member of a practice’s staff recommending to a patient that they use an online service and showing them how to do that. A poster in a waiting room, or a message played to callers to the practice’s telephone line, is passive facilitation. Helping patients only when they ask for assistance is reactive, whereas offering help before it is sought is proactive. Our literature review found that most published studies concern active digital facilitation, while respondents to our survey of GP practices more commonly reported passive and reactive approaches rather than active and proactive, and this was borne out by the qualitative study. The survey of patients demonstrated low awareness of any type of digital facilitation taking place, alongside an often-unfulfilled wish for proactive support to use online services.

##### Theme 6

Supporting *initial sign-up to online services versus sustained use* thereafter: the literature we reviewed implies that most digital facilitation is focused on achieving initial patient sign-up, rather than supporting sustained use thereafter. The questions we asked in the practice survey were about digital facilitation in general and did not ask separately about support for ongoing use of online services as compared with supporting initial registration to use those services. Our qualitative research findings, added at the second synthesis workshop and confirmed at the third, are consistent with this: digital facilitation by primary care practices more often concerns getting people signed up rather than providing ongoing support to patients subsequently. The patient survey results, discussed at the third synthesis workshop, showed that initial registration for an online service in general practice can be a hurdle for patients.

##### Theme 7

*Who delivers the digital facilitation* was not a focus of the literature found in our review. However, the examples in the literature mostly refer to primary care physicians and nurses helping with digital facilitation. Our survey of practices found that GPs and practice staff view the responsibility for digital facilitation as shared between practices and other parts of the NHS regionally and nationally, and that within practices it is the administrative staff who provide most support. Our qualitative research, discussed at the second and third synthesis workshops, found a “bystander effect”: different staff groups, patients, and other stakeholders identify other groups as being responsible for digital facilitation. NHS stakeholders at national and regional levels placed responsibility with clinicians and other practice staff. Primary care clinicians seem to place responsibility on practice reception staff (and on patients to sort themselves out); patients viewed it as the responsibility of the NHS more widely.

##### Theme 8

*Enablers of digital facilitation* were referred to in many papers in the literature we reviewed. Enablers include staff and patients’ perceptions of the usefulness of the online services, along with the user-friendliness of the digital platforms that patients are to use. The practice survey results, discussed at the first synthesis, found that the majority of practices had positive perceptions of online services (that they lead to operational efficiencies). The qualitative exploration found other enablers of digital facilitation, all also mentioned in the literature, including funding or paid time for staff to deliver the facilitation, expectation that an online service will be useful to patients or bring operational efficiencies for the practice or both, the existence of guidelines, 1 or more of the practice staff having specific responsibility for digital facilitation, and patients’ trust in practice staff. The patient survey found that only 13% (392/2935) of patients reported having been given help to use online services but also yielded suggestions for enabling digital facilitation.

##### Theme 9

*Barriers to digital facilitation* can include staff attitudes toward online services and stereotyped assumptions about the capabilities of some patients, such as the elderly. The multiplicity of different platforms for online services makes digital facilitation more difficult. The literature review highlighted the need for staff time and capacity to deliver digital facilitation, and the practice survey found that most practices consider that they lack the staff time and ability to sufficiently support patients in using online services. Our qualitative research confirmed the existence of all these barriers and also found that digital facilitation can be a low priority for practice staff. The patient survey found that some patients are unaware of online services and others are unhappy about them and resist using the services regardless of facilitation efforts.

##### Theme 10

*Inequalities in digital access and digital facilitation* between subgroups in the population: we found little information in the literature about how well digital facilitation works for different population subgroups, although some literature pointed out the risk of practice staff concentrating on the most digitally literate patients and neglecting others who might be in more need of support. In our practice survey, we asked whether digital facilitation was particularly focused on any subgroups of the population. Practices responding frequently reported targeting digital facilitation at older adults. The qualitative research reinforced that a patient’s age may be assumed (not necessarily correctly) by staff to be an indicator of digital competence. But many practices also reported “targeting” all the other patient subgroups suggested in the questionnaire, which calls into question what such “targeting” amounts to. The patient survey found that older patients were less likely to be aware of or use digital facilitation.

##### Theme 11

*Effectiveness of digital facilitation*: the literature review revealed examples of where digital facilitation had successfully encouraged initial registration with online services, including when GPs and other practice staff personally recommend online services and when practices run introductory sessions for patients. Ongoing guidance and support that is delivered within primary care consultations may be effective not only in increasing initial registration with online services but also in sustaining their subsequent use [12]. We did not address this theme in our survey of practices, which was more concerned to identify the extent and type of digital facilitation activities being undertaken, and enablers and barriers for such activities. The qualitative exploration, discussed at the second synthesis workshop, found that some practices highlight how many patients sign up to online services as evidence of effectiveness—but without knowing the contribution to that of digital facilitation efforts. Our patient survey findings, discussed at the third (final) synthesis workshop, suggest that practices that report using displays, social media, workshops, or events for digital facilitation were more likely to have patients who are aware of and use digital facilitation. We found no such difference, however, for practices that use leaflets, text messages (or emails), or online approaches to digital facilitation for use of online services. Practices that use a “practice champion” for online services have more patients who report being told about such services than other practices.

### Program Theory

From the findings of our research as captured in the 11 themes, we developed the program theory using a realist approach to describe provision of digital facilitation in terms of the context in which it takes place, the flow of activities comprising the intervention, and the theory and assumptions underlying the intervention, including the intended outcomes.

Digital facilitation in primary care in England takes place in a context of NHS policy to encourage greater use of digital services, which are seen as a way to improve patients’ access to care, improve triage systems, and streamline service delivery. But our research found that practice staff and patients do not always share this view of the value of online services, which makes it challenging to facilitate their use. The context of digital facilitation is also characterized by a multiplicity of platforms for delivering online services. Individual GP practices choose which to use, and the result is a patchwork of different systems and approaches. The COVID-19 pandemic and the measures taken in response greatly accelerated the introduction and use of online services. Our qualitative research found that at the same time some practices stopped providing digital facilitation activities during the pandemic and did not resume them.

The activities that could comprise digital facilitation are varied. Active forms of facilitation include practice champions, training, and workshops. Passive forms include informational leaflets, text messages, and recorded messages on practices’ telephone lines. We found that most facilitation is reactive, relying on the digital skills of practice staff—especially reception and administrative staff—to respond to a patient’s immediate needs, rather than forming part of a wider effort to enable participation in a health service that increasingly relies on online services. There is evidently some confusion over who has responsibility for supporting patients to use online services. Although practice staff are largely undertaking the digital facilitation that is taking place, they and patients saw a role also for other parts of the NHS at regional and national levels, for example, to tackle the need for wider efforts to educate patients about the benefits of booking appointments online.

Digital facilitation can help patients both directly—to access services such as ordering repeat prescriptions online —and indirectly—by making them more confident users of online services. Our research has shown that the pathways linking digital facilitation to expected beneficial outcomes can be complex. Patients want help to access care and not necessarily help with online services. It is, therefore, unsurprising that we found that digital facilitation is frequently just responding to immediate issues of patient access rather than being aimed at building patients’ capacity to access and continue to use online services generally. The path from digital facilitation to its hoped-for benefits requires not only that patients sign up to online services but also that they continue to use them thereafter. Digital facilitation needs to be focused on the latter rather more than is currently the case. Digital facilitation could also be more responsive to inequalities in accessing NHS online services. Our survey showed that practices are aware of the need particularly to support older age groups, non-White ethnicities, lower socioeconomic groups, those in poorer health, and those in rural settings, who may struggle to access digital services. But it was unclear in what ways digital facilitation was being tailored for such patients. Our survey of patients found that ethnic minorities and those for whom English is not a first language are more likely to be aware of and use digital facilitation. However, we also found that older patients are less likely than others to be aware of, or make use of, digital facilitation and (with assumptions made by staff about the impact of older age) are less likely to be told about digital services or helped to use them.

### Implementation Theory

Implementation of digital facilitation varies across general practices, both in the capacity to provide it and in the types of facilitation used. Practice populations also differ—for example, in health needs, age structure, and ethnicity—and hence have varying needs for digital facilitation. Within themes 8 and 9 we describe the range of enablers of, and barriers to, implementing digital facilitation. The staff time and resources available to a practice clearly are a major constraint. Most practices we surveyed felt that they had insufficient capacity to provide digital facilitation to the extent they would like. Reactive and passive approaches are more commonly used and they require less staff time (at least in the short term) than more active approaches.

The quality and usefulness of online services as perceived by practice staff and patients affect how, and how far, digital facilitation is implemented. Online services that are easy to use and with clearly apparent benefits require less facilitation effort. For example, repeat prescription ordering online was found in our patient survey and qualitative case studies to be relatively well used. Our qualitative research indicated that issues with more difficult to use services are seen by staff as not their responsibility to resolve. The diverse and changing mix of online services not only presents challenges to patients in understanding what is available and with what support but can also create issues for staff in maintaining knowledge of the online services and the requisite skills to provide facilitation.

Unclear lines of responsibility can also hinder implementation. Practice staff, patients, and other stakeholders (suppliers of digital technology; other parts of the NHS) may each assume that some responsibility for digital facilitation lies with other parties. When no one considers it their responsibility to support patients with broader issues of digital access such as digital literacy and confidence, then these aspects are likely to be neglected. In general, implementation would be aided by all members of staff in a general practice having a clear, shared understanding of what digital facilitation its patients need and how to deliver that.

### Evaluation Framework

One aim of our research was to design a framework for future evaluations of the effectiveness and costs of digital facilitation interventions. Based on our synthesis of the findings from all 4 work packages, we propose an evaluation framework consisting of 4 aspects. The high-level nature of the proposed framework reflects what we have learned about the awareness and extent of digital facilitation in the NHS in England. The implementation of future evaluations will require engagement with all stakeholders and with policy makers. Within the bounds of timescales and funding available, an iterative approach to evaluation, with progressive and cumulative learning, is, as ever, desirable.

1. Digital facilitation as an intervention: digital facilitation may be defined as support to enable patients to achieve access to care services digitally. Many types of digital facilitation interventions are possible, are not mutually exclusive, and are often complex [15].
2. Responsibility for digital facilitation: clarity about who is responsible for (which) digital facilitation is key to it happening: which practice staff are responsible and how this fits with their role; what is expected of patients; are any third parties (eg, technology suppliers and charities working with patients) involved in delivering or supporting digital facilitation and how do they interface with practice staff and patients; and how does digital facilitation fit into the wider health care community beyond general practice?
3. Patient groups and potential for inequalities: for which groups of patients is digital facilitation most needed; do such groups differ in the extent to which they would benefit from, or be burdened by, online services and will they have different views of the importance of online services; and which types of facilitation are most effective for which groups?
4. Outcome and cost measures: Potential measures of outcomes (intended and unintended) include the following and should be collected in a way that permits determination of inequalities between subgroups in the population: patient awareness of, registration with, and sustained use over time of digital services; measures of access to GP services and rates of digital access within that; patient-reported experiences of engaging with facilitation and of the online services used; practice staff–reported experiences with facilitation (ease of delivery, impact on workload, and views on whether it is working); costs to general practices of digital facilitation (training, staff time, materials, and equipment); costs or savings to practices from changed use of online services by patients; costs or savings to the rest of the health care system; and costs or savings (money and time) to patients from using online services. Within the bounds of feasibility and funding, the longer the period over which outcome and cost data can be collected the more complete will be the understanding of longer-term impacts.

## Discussion

### Principal Findings

To the research team’s knowledge, our research is the first to explore the range and extent of digital facilitation in primary care in a health care system. We have found that the digital facilitation being provided by general practices varies from place to place but is often passive and reactive, rather than active or proactive, and is focused on immediate difficulties rather than on broader and longer-term support for patients generally to use online primary care services. By identifying themes emerging from our findings when taken together, we have developed a program theory and an implementation theory of digital facilitation in primary care, which in turn have enabled us to construct a framework for future evaluations. These lead collectively to the following implications for policy, practice, and research.

### Implications for Policy

A range of policy implications is evident and may well apply in many other countries than just England. Digital facilitation has a role in achieving the move to greater use of digital services in health care. But there is a disconnect between policy makers’ expectations about what use of online services might achieve and the limited efforts at digital facilitation that are occurring at general practice level. A first step for policy makers is to recognize the existence of this disconnect. Rectifying it implies a need to better articulate to service users and practice staff the hoped-for benefits of the online services. To deliver digital facilitation requires investment in staff time and training. It also requires clarity about how the responsibility for supporting patients to use online services is distributed across different parties. Although some responsibility for digital facilitation falls on practice staff, there is a role for complementary support from health service organizations nationally or regionally [16], and this needs to be recognized and acted upon. An additional option is for the providers of online service platforms to be encouraged, or mandated, to offer support to patients either directly or by helping general practice staff to deliver digital facilitation (see, for example, eConsult [17]).

### Implications for Practice

There is clear potential for digital facilitation to help patients and practices use online services. Realizing this potential requires investment in, as well as by, general practices. It also requires clear, shared understanding of which staff are responsible for doing what [18]. Practice leaders and managers need to take responsibility for their practice’s digital facilitation strategy and associated training and resources being provided for those staff tasked to deliver facilitation activities [19]. There exists a wide range of facilitation types and it is likely that a combination of approaches would be appropriate. Attention needs to be paid to the likely differing digital facilitation needs of subgroups of the patient population, rather than assuming that a generic approach will suffice. Getting patients signed up with online services is a necessary but insufficient condition for realizing the benefits of the services. Monitoring and support for continued use after initial sign-up are also needed but appear to receive insufficient attention currently. Finally, it is inevitable that some patients will never be able or willing to use some (or any) online services; hence adequate, equitable, and nondigital access to care will need to remain an option however good digital facilitation becomes.

### Implications for Research

There is great scope for useful research around digital facilitation. There is a need to explore the association between patients’ awareness of online services and their use of them, and how different digital facilitation approaches affect that. Measuring the effectiveness of digital facilitation efforts requires a holistic approach in line with guidance on complex interventions, which suggests that evaluation goes beyond whether an intervention works [15]. Our research suggests several areas for future evaluation of digital facilitation, where current evidence is lacking, including:

- the extent of patient demand for online services to access general practice, and how this varies between population subgroups,
- the level and mix of digital facilitation interventions needed,
- the role of general practice administrative staff in supporting online services,
- the relative effectiveness and costs of different approaches to digital facilitation,
- how different approaches to digital facilitation work for different population subgroups, and
- focusing on supporting sustained patient use of online services, not just initial registration.

### Strengths and Limitations

A strength of the research project was the collection of evidence from 4 main sources and the triangulation between them that enabled. The 4 work packages were deliberately staggered and yielded results at different times. We exploited that through a cumulative design of the evidence synthesis, comprising 3 workshops (as successive work packages yielded findings) and a final stakeholder challenge meeting, which took place at intervals over 13 months. This design was chosen to ensure a thorough, effective, and efficient process with time for detailed challenge and discussion. Members of the public were actively involved throughout, including at the 3 synthesis workshops, and they were supported throughout by a researcher in our team specializing in that role. The public participants made pertinent challenges and shared experiences in each workshop leading to clearer thinking about the context and meaning of the research findings.

The principal limitation of the synthesis reported here is that it is based on evidence gathered during a period of great change in primary care in the United Kingdom (as elsewhere): 2020‐2022. The COVID-19 pandemic prompted great and sudden changes in the practice of primary care, with much greater emphasis on providing services remotely rather than in person. The full implications of those changes are not yet wholly apparent. We have noted their importance and taken them into account to the extent possible so far.

### Conclusions

Digital facilitation is important in the context of increasing opportunities for online access to services in primary care. It can take many forms, though much of what is currently done in GP practices in England is reactive and passive. There is scope to develop an approach to facilitation that more actively engages patients. There seems to be a disconnect between stakeholders’ expectations and perceptions of how digital facilitation could help and the reality seen in everyday practice. Digital facilitation requires staff time and resources, along with clarity over responsibilities. The establishment of clear lines of responsibility, the development of digital tools and platforms that work well for patients and practice staff, and investment in staff time and training will all be needed if digital facilitation is to deliver on its promise. Based on synthesis of the findings from our research, we propose a framework for future evaluations of the effectiveness and costs of digital facilitation interventions.

## Abbreviations

GP: general medical practitioner (primary care physician)
NHS: National Health Service (UK)
